# Hierarchical Organization of Multi-Site Phosphorylation at the CXCR4 C Terminus

**DOI:** 10.1371/journal.pone.0064975

**Published:** 2013-05-29

**Authors:** Wiebke Mueller, Dagmar Schütz, Falko Nagel, Stefan Schulz, Ralf Stumm

**Affiliations:** Institute of Pharmacology and Toxicology, Jena University Hospital, Friedrich Schiller University, Jena, Germany; Washington University, United States of America

## Abstract

The chemokine receptor CXCR4 regulates cell migration during ontogenesis and disease states including cancer and inflammation. Upon stimulation by the endogenous ligand CXCL12, CXCR4 becomes phosphorylated at multiple sites in its C-terminal domain. Mutations in the CXCR4 gene affecting C-terminal phosphorylation sites are a hallmark of WHIM syndrome, a genetic disorder characterized by a gain-of-CXCR4-function. To better understand how multi-site phosphorylation of CXCR4 is organized and how perturbed phosphorylation might affect CXCR4 function, we developed novel phosphosite-specific CXCR4 antibodies and studied the differential regulation and interaction of three C-terminal phosphorylation sites in human embryonic kidney cells (HEK293). CXCL12 promoted a robust phosphorylation at S346/347 which preceded phosphorylation at S324/325 and S338/339. After CXCL12 washout, the phosphosites S338/339 and S324/325 were rapidly dephosphorylated whereas phosphorylation at S346/347 was long-lasting. CXCL12-induced phosphorylation at S346/347 was staurosporine-insensitive and mediated by GRK2/3. WHIM syndrome-associated CXCR4 truncation mutants lacking the S346/347 phosphosite and the recently identified E343K WHIM mutant displayed strongly impaired phosphorylation at S324/325 and S338/339 as well as reduced CXCL12-induced receptor internalization. Relevance of the S346-S348 site was confirmed by a S346-348A mutant showing strongly impaired CXCL12-promoted phosphorylation at S324/325 and S338/339, defective internalization, gain of calcium mobilization, and reduced desensitization. Thus, the triple serine motif S346-S348 contains a major initial CXCR4 phosphorylation site and is required for efficient subsequent multi-site phosphorylation and receptor regulation. Hierarchical organization of CXCR4 phosphorylation explains why small deletions at the extreme CXCR4 C terminus typically associated with WHIM syndrome severely alter CXCR4 function.

## Introduction

Stimulus-dependent phosphorylation of G protein-coupled receptors (GPCRs) represents a major mechanism regulating signal transduction and receptor trafficking [1], [2]. Most GPCRs contain multiple phosphorylation sites and a range of protein kinases, activated by distinct mechanisms, are able to phosphorylate GPCRs [3]. Thus, multi-site phosphorylation of a GPCR permits the integration of distinct influences and tissue-specific control of signaling processes but also raises the question whether different phosphosites are redundant in their function.

G protein-coupled CXCR4, the receptor of the chemokine CXCL12, is essential for embryonic development of various organ systems, serves as HIV coreceptor, and plays a major role in diseases including cancer and inflammation [4], [5], [6], [7], [8], [9], [10], [11]. CXCR4 contains 18 potential serine/threonine phosphorylation sites in the C-terminal domain [12]. CXCL12-induced phosphorylation has been confirmed for 6 of these sites by tandem mass spectrometry and phosphosite-specific antibodies [13], [14]. The different phosphosites are phosphorylated by various kinases including G protein-coupled receptor kinases (GRKs) and protein kinase C (PKC) [14]. Depending on the cell type, also heterologous stimuli like the phorbol ester PMA and epidermal growth factor (EGF) cause CXCR4 phosphorylation [12], [13], [14], [15]. Ranging from S324 to S352, the confirmed phosphosites are dispersed over a large part of the CXCR4 C terminus [14]. The different sites exert distinct functions in uncoupling activated CXCR4 from G protein, stimulus-induced internalization and degradation, as well as G protein-independent signaling of CXCR4 [14], [16], [17], [18], [19]. Consistently, experimental manipulations of GRKs and PKC isoforms proved that interfering with the phosphorylation machinery perturbs signaling and regulation of CXCR4 and eventually leads to aberrant cell migration and tumor growth [15], [20], [21], [22], [23], [24], [25], [26].

Early reports analyzing CXCR4 C-terminal truncation mutants described that deletions within the 12 C-terminal residues severely impair CXCL12-induced internalization – an effect expected to reduce CXCR4 desensitization [16], [17]. Consistently, mutations in the human CXCR4 gene causing small C-terminal deletions of 10 to 19 residues or a single amino acid exchange (E343K) are associated with WHIM syndrome (a rare immunodeficiency characterized by warts, hypogammaglobulinemia, infections, and myelokathexis) and gain-of-CXCR4-function [27], [28], [29], [30]. The causal connection between WHIM syndrome and dysregulated CXCR4 signaling has been confirmed in *knockin* mice carrying a heterozygous R334X truncation mutation, which corresponds to the most frequent WHIM syndrome-causing mutation in humans [31].

Thus, while phosphorylation occurs at multiple dispersed CXCR4 C-terminal sites [14], small mutations close to the CXCR4 C-terminal end are sufficient for severely perturbed CXCR4 function [16], [17], [29], [30]. In the last few years, our group and other laboratories identified examples for hierarchical GPCR phosphorylation in which a single site dictates whether or not other phosphosites become efficiently phosphorylated [3], [32]. Given the severe effect of the WHIM mutations, we hypothesized that the last 10 CXCR4 residues contain a major phosphosite that cooperates with the more N-terminal regulatory elements. To test this hypothesis, we generated novel phospho-selective CXCR4 antibodies and examined site-specific phosphorylation at S324/325, S338/339, and S346/347 in CXCR4 wildtype and WHIM syndrome mutant receptors. We provide evidence that serine residues 346/347 represent a rapidly phosphorylated major site and that the S346-S348 motif is necessary for efficient subsequent phosphorylation at residues S324/325 and S338/339 as well as CXCR4 internalization.

## Materials and Methods

### Compounds

CXCL12 (PeproTech, Hamburg, Germany),^ 125^I-CXCL12 (Hartmann Analytic, Braunschweig), AMD3100 (#A5602, Sigma-Aldrich), Lambda Protein Phosphatase (#P0753, New England Biolabs GmbH, Frankfurt am Main, Germany), phorbol 12-myristate 13-acetate (#P1585, Sigma Aldrich), forskolin (#P6886, Sigma Aldrich), epidermal growth factor (AF-100-15, PeproTech, Hamburg, Germany), staurosporine (#S-4400, Sigma Aldrich).

### Antibodies

Phosphosite-specific rabbit antibodies for the S338/339-phosphorylated and S346/347-phosphorylated forms of the CXCR4 receptor were generated against the following peptides: RGGH(pS)(pS)VSTE (S338/339) and VSTESE(pS)(pS)SFHSS (S346/347), which contained two phosphorylated serine residues (pS). The peptides were purified by HPLC, coupled to keyhole limpet haemocyanin and mixed 1∶1 with Freund’s adjuvant for immunization. Specificity of the antisera was tested using dot blot analysis. For subsequent analysis, antibodies were affinity purified against their immunizing peptide and against the non-phosphorylated peptide using the SulfoLink kit (Thermo Scientific, Rockford, IL). The polyclonal phosphosite-specific antibody for the S324/325-phosphorylated form of the CXCR4 receptor (#5199) was a kind gift of Dr. Jeffrey L. Benovic [14]. T7 epitope tag was detected using affinity-purified anti-T7 antibody (Gramsch Laboratories, Schwabhausen, Germany). The rabbit anti-HA antibody and the UMB-2 anti-CXCR4 antibody have been described [33], [34], [35]. Anti-transferrin receptor antibody was purchased (#13-6800; Life Technologies, Darmstadt, Germany).

### Plasmid Constructs

cDNA for wildtype CXCR4 (CXCR4-WT) was amplified by reverse transcription PCR from mouse spleen cDNA with Nhe I/Not I extensions. The construct received an N-terminal HA epitope tag or an N-terminal T7 epitope tag and was subcloned into pcDNA^TM^3.1(+) vector (#V790-20, Life Technologies). The cDNA for a CXCR4-ST/A mutant lacking C-terminal serines and threonines, a S346-348A mutant, an E343K mutant, and for CXCR4 mutants lacking the last 10 and 17 residues (CXCR4-Δ10, CXCR4-Δ17) were generated by gene synthesis (Eurofins MWG Operon, Ebersberg, Germany) and inserted into CXCR4-WT using the endogenous Sph I site (bp 967) and the Not I extension. Sequence identity was verified by double strand sequencing.

### Cell Culture and Transfection

Human embryonic kidney cells (HEK293 cells, DSMZ, Braunschweig, Germany) were cultivated in DMEM (PAA Laboratories, Pasching, Austria) supplemented with 10% defined fetal bovine serum (FBS GOLD, PAA), 2 mM L-glutamine, and 100 units/ml penicillin/streptomycin (PAA). JetPEI reagent (PEQLAB Biotechnology, Erlangen, Germany) was used for transient and stable transfections. Stable transfectants were selected in the presence of 400 µg/ml G418.

### Radioligand Assays

Binding and internalization assays were performed as described [35] using 25 pmol/l ^125^I-CXCL12. For homologous competitive radioligand binding, cells were incubated for 2 h at 4°C with ^125^I-CXCL12 containing increasing amounts of unlabeled CXCL12. Cells were washed twice with ice cold phosphate buffered saline (PBS) and then lysed in 10 mM Tris buffer (pH7.4). IC_50_ was calculated using the “homologous competitive binding curve” option of the GraphPad Prism 4.0a software. For pulse chase analyses of radioligand/receptor internalization, cells were loaded with ^125^I-CXCL12 for 2 h at 4°C. Cultures were washed with ice cold PBS and harvested either immediately (starting value) or transferred to 37°C to permit internalization. Residual surface-bound ^125^I-CXCL12 was stripped by a double wash with acidic citrate buffer. Then, cells were lysed in 10 mM Tris buffer. Counts of mock-transfected cultures were subtracted.

### Immunocytochemistry and ELISA

Cells were processed as described [35]. Briefly, T7-N-tagged or HA-N-tagged surface receptors were pulse-labeled in life cells at 4°C with anti-tag antibody. For immunocytochemistry, cultures were washed with PBS after 30 min, supplemented with medium containing CXCL12 or vehicle, and placed at 37°C for 30 min. Cells were fixed, permeabilized, and detected using Cy3-AffiniPure goat anti-rabbit IgG (#111-165-003, Jackson ImmunoResearch Laboratories, Suffolk, United Kingdom). Labeling was imaged using a LSM 510 Meta confocal laser scanning microscope (Carl Zeiss, Jena, Germany). For ELISA, cultures were washed with PBS after 60 min, fixed and detected as described [35].

### Immunoblotting

Immunoblotting procedures were performed as described [34], [35] using 2,000,000 HEK293 cells. Cells were starved in DMEM for 3 h before they were stimulated. For CXCL12-washout experiments, an acidic buffer (50 mM sodium citrate, 90 mM NaCl, pH4.5) was applied before cells were supplemented with CXCR4 antagonist-containing DMEM (AMD3100; 6 µM). Cells were lysed in 1 ml RIPA buffer and centrifuged for 30 min at 23,000×g at 4°C. Thereafter, the protein concentration was measured in the supernatants (Pierce 660 nm Protein Assay). Equal amounts of protein were used. Glycoproteins were enriched using wheat germ lectin agarose beads. For dephosphorylation, beads were incubated for 3 h at 37°C with Lambda-Protein Phosphatase. Beads were washed with RIPA buffer before proteins were eluted for 20 min at 60°C with SDS sample buffer. Samples were then subjected to 10% SDS-polyacrylamide gel electrophoresis and immunoblotted. Blots were immunodetected using anti-CXCR4 (UMB-2), anti-pS338/339 (#3152), anti-pS346/347 (#3208), anti-pS324/325 (#5199), anti-HA-tag (#631), anti-T7-tag or anti-transferrin receptor antibodies and appropriate peroxidase-conjugated secondary antibodies (#sc2004, Santa Cruz Biotechnology Inc., Heidelberg, Germany; #NXA931 GE Healthcare). Signals were imaged and analyzed using the Fusion-FX7 Chemiluminescence System and BIO-1D software (PEQLAB). For quantitative analysis of the signals of the phospho-sensitive antibodies (UMB-2, #3152, #3208, #5199) each signal was divided by its anti-HA signal. Further normalizations were performed as described in *Results*.

### RNA Interference

An established siRNA-mediated knockdown protocol for GRK2 and GRK3 was used [36]. The following siRNAs (Dharmacon, Hilden, Germany) were transfected with HiPerFect (#301707, Qiagen): GRK2 (5′-CCGGGAGAUCUUCGACUCAUAdTdT-3′), GRK3 (5′-AAGCAAGCUGUAGAACACGUAdTdT-3′), non-silencing control (5′-GCUUAGGAGCAUUAGUAAA-3′). Cells were assayed 3 days after transfection. Silencing was quantified by immunoblotting using anti-GRK2 and anti-GRK3 antibodies sc-562 and sc-563. (Santa Cruz). All experiments showed protein level reductions of approximately 80%.

### [Ca^2+^]_i_ Measurements

Cells were co-transfected with CXCR4 constructs and a 16z44 G protein chimera engineered to link G_i/o_-coupled receptors to Ca^2+^ mobilization [37]. CXCL12 was administered and [Ca^2+^]_i_ measured using the FlexStation3 microplate reader as described [35]. For single stimulation, CXCL12 was applied to a final concentration of 80 nM. In sequential stimulation experiments, a second CXCL12 stimulus was applied 90 s after the first CXCL12 stimulus and recording was continued for 120 s.

### Statistics

Statistical tests were done by ANOVA including the indicated post-tests or by Student's t-test using GraphPadPrism software.

## Results

### Characterization of Phospho-sensitive Anti-CXCR4 Antibodies

To investigate multi-site phosphorylation properties of CXCR4, we generated two novel polyclonal phospho-selective antibodies for S338/339 and S346/347 and used them along with a polyclonal antibody for phosphorylated S324/325 [14] and the monoclonal antibody UMB-2, which recognizes only the non-phosphorylated C terminus [33], [34]. The four antibodies were tested in dot blots with serial dilutions of phospho- and non-phosphopeptides corresponding to 3 different segments between CXCR4 C-terminal residues 322 and 352 (Figure S1A). The assay identified the last 12 C-terminal residues as the UMB-2 epitope and showed that UMB-2 binds the epitope only when serine residues 346/347 are not phosphorylated (Figure S1). The three phospho-selective antibodies were specific for their cognate phosphorylated epitope and showed minimal cross-reactivity to the other phospho- or non-phosphoepitopes (Figure S1B). We therefore refer to UMB-2 as anti-S341-S352 and to the phospho-selective antibodies as anti-pS324/325, anti-pS338/339, and anti-pS346/347.

**Figure 1:**
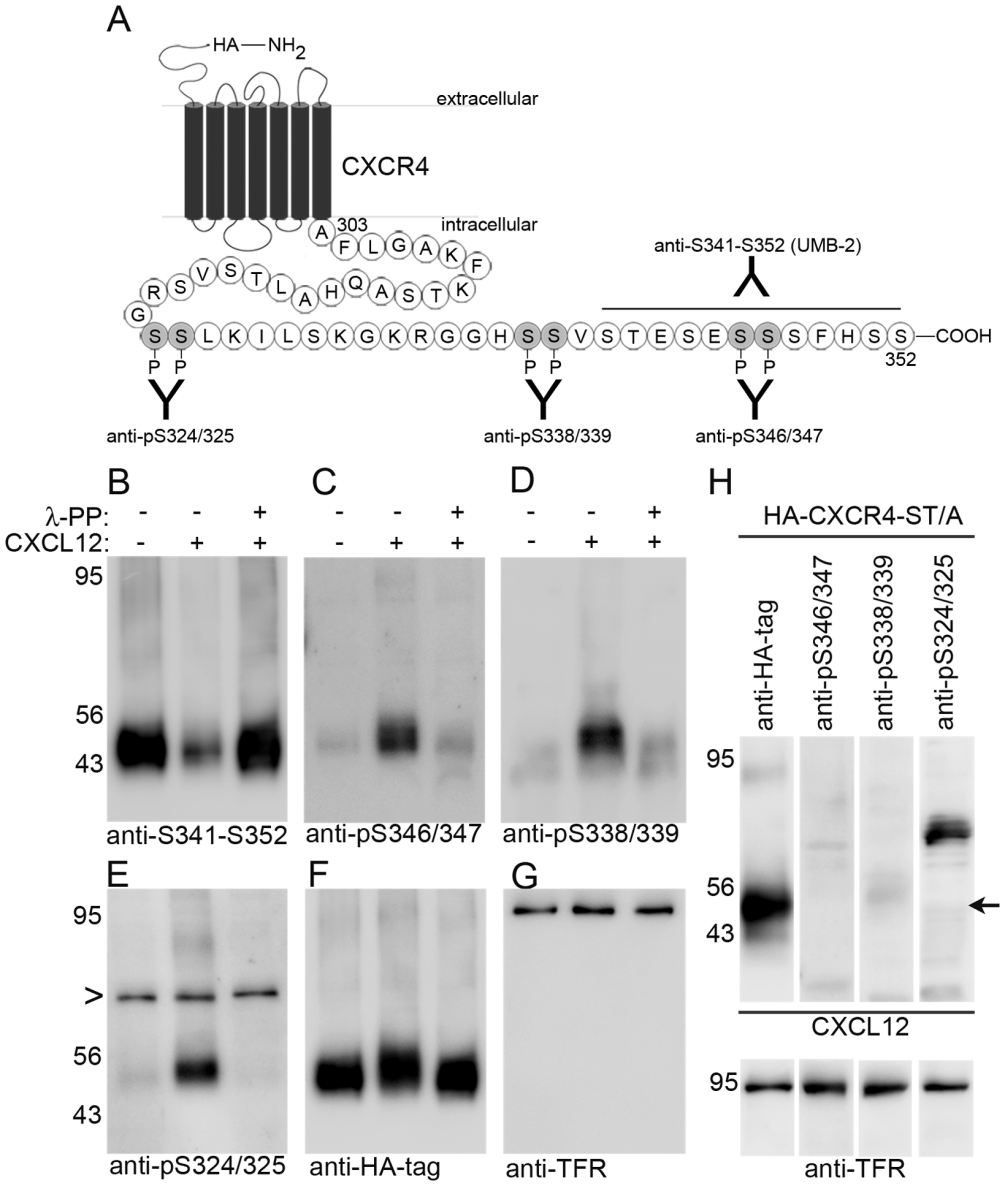
Specificity of phospho-sensitive anti-CXCR4 antibodies. **A**, Schematic representation of the anti-pS324/325, anti-pS338/339, anti-pS346/347, and anti-S341-S352 (UMB-2) antibodies with their epitopes in the human CXCR4 receptor. **B–G**, HEK293 cells stably transfected with hemagglutinin (HA) epitope-tagged CXCR4 received CXCL12 (20 nM) or vehicle for 15 min and were lysed. An aliquot of the lysate from stimulated cells was dephosphorylated using Lambda-Protein Phosphatase (λ-PP). Samples were separated on 10% SDS polyacrylamide gels, blotted, and detected with anti-S341-S352 (B), anti-pS346/347 (C), anti-pS338/339 (D), anti-pS324/325 (E), anti-HA-tag (F), and anti-transferrin receptor (TFR) (G). **B**, CXCL12 stimulation causes a strong loss of anti-S341-S352 binding; dephosphorylation restores antibody binding. **C–E**, The phospho-selective antibodies recognize CXCR4 only in the non-dephosphorylated sample from stimulated cells. Anti-pS324/325 produces a non-specific band at approximately 70 kDa (arrowhead in E). **H**, HEK293 cells transiently transfected with a HA-CXCR4-ST/A mutant (all serines and threonines in the C-terminal domain were converted into alanines) were stimulated with CXCL12. Four aliquots were loaded in a Western blot and separately detected with the indicated antibodies. The phospho-selective antibodies do not detect the CXCL12-stimulated phosphorylation-deficient CXCR4 mutant (the arrow points to 47 kDa which corresponds to the size of CXCR4). **F**,**G**,**H**, Equal loading was controlled in stripped blots by detecting the HA-tag of recombinant expressed CXCR4 and by detecting endogenous TFR.

The four antibodies were then characterized in immunoblots with lysates from HEK293 cells stably expressing CXCR4 with an N-terminal hemagglutinin (HA) epitope tag. The cultures received vehicle or CXCL12 for 15 min before they were harvested. Aliquots of CXCL12-stimulated lysates were dephosphorylated with Lambda-Protein Phosphatase (λ-PP) before loading. Anti-S341-S352 detected a broad band of the expected size at 47 kDa. The signal was strongly reduced after CXCL12-stimulation and was fully recovered after dephosphorylation (Figure 1B). Thus, while anti-S341-S352 fails to detect C-terminally phosphorylated/activated CXCR4 in non-dephosphorylated samples, it detects total CXCR4 in dephosphorylated samples.

The phospho-selective antibodies produced strong CXCR4-specific signals in CXCL12-stimulated samples and weak signals in non-stimulated and dephosphorylated samples (Figure 1C–E). CXCL12 stimulation of HEK293 cells transiently transfected with a phosphorylation-deficient HA-tagged CXCR4 mutant (HA-CXCR4-ST/A) did not result in phospho-selective signals (Figure 1H). Similar amounts of total CXCR4 and equal loading were confirmed for all samples by detecting total CXCR4 with anti-HA antibody and by detecting endogenous transferrin receptor (TFR) (representative examples are shown in Figure 1F–H). In conjunction with the dot blot epitope mapping (Figure S1) these experiments demonstrate that the four antibodies are specific for CXCR4 and that anti-S341-S352 (UMB-2) recognizes the non-phosphorylated 12 C-terminal residues while anti-pS324/325, anti-pS338/339, and anti-pS346/347 are selective for the respective phosphorylated sites. Analyses of non-transfected HEK293 cells showed that anti-S341-S352 readily detected endogenous CXCR4 whereas the phospho-selective antibodies failed to produce signals under these conditions (not shown). This indicates that the sensitivity of anti-S341-S352 is considerably higher than that of the phospho-selective antibodies.

### CXCL12 Induces Sequential Multi-site Phosphorylation at the CXCR4 C Terminus

**Figure 2:**
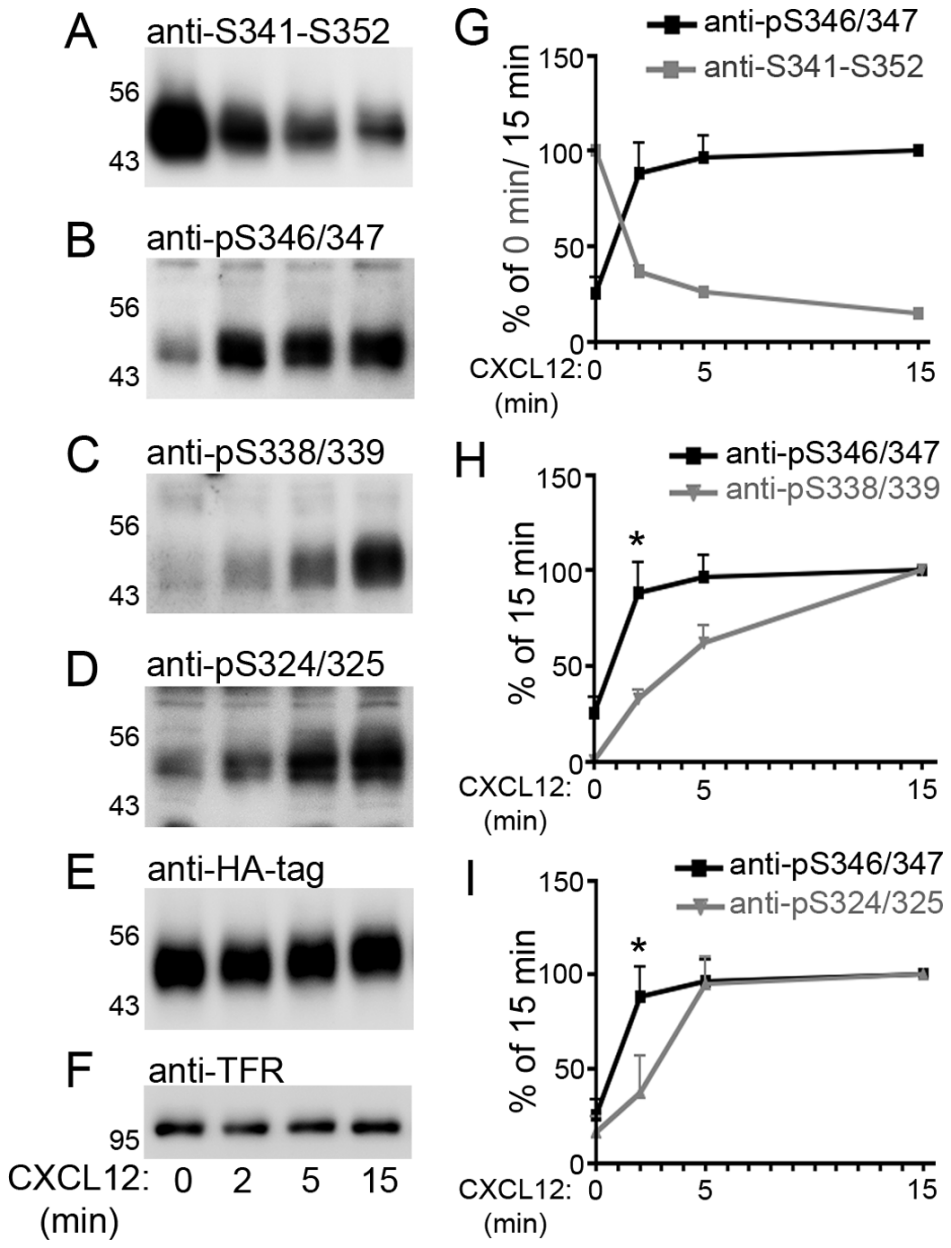
**Sequential phosphorylation at CXCR4 C-terminal sites.** **A–F**, Stably CXCR4-transfected HEK293 cells were stimulated with 20 nM CXCL12 for 0, 2, 5, and 15 min. Aliquots of the lysates were detected in four immunoblots using the indicated antibodies. Blots were stripped and reprobed with anti-HA and anti-transferrin receptor (TFR) to control for equal loading. **A**,**B**, CXCL12 induces a rapid loss of the anti-S341-S352 signal and a concomitant immediate increase of the anti-pS346/347 signal. **C**, Maximal S338/339 phosphorylation requires 15 min CXCL12 stimulation. **D**, Demonstration of rapid CXCL12-promoted S324/325 phosphorylation. **E**–**I**, The signal ratio versus anti-HA was determined for the four anti-CXCR4 antibodies using densitometry. The ratio was set as 100% at 0 min for anti-S341-S352 (G) and at 15 min for the phospho-selective antibodies (G–I). Data represent mean+SEM from 4–5 independent experiments. **G**, Note complementarity of the anti-S341-S352 and anti-pS346/347 time courses. **H**,**I**, Phosphorylation occurs faster at S346/347 than at S338/339 and S324/325. *: p<0.05; 1way ANOVA and Bonferronís multiple comparison post-test for selected groups.

We then used the four antibodies to examine kinetics of CXCL12-induced phosphorylation. Stable CXCR4 transfectants were harvested immediately before as well as 2, 5, and 15 min after CXCL12 stimulation. Aliquots of the lysates were loaded four times, immunoblotted, and detected with anti-S341-S352 and the three phospho-selective antibodies (Figure 2A–D). Blots were stripped and reprobed with anti-HA to detect total CXCR4 and with anti-TFR as additional loading control (Figure 2E,F). Signal intensities were measured by CCD camera-based densitometry. For each sample, the signals obtained with the four phospho-sensitive antibodies were divided by the signal obtained with the anti-HA antibody before independent repeats were averaged. In the case of anti-S341-S352, the CXCL12-induced signal loss was determined by expressing intensities of CXCL12-stimulated samples as percentage of the non-stimulated sample (Figure 2G). In the case of the three phospho-selective antibodies, the CXCL12-induced signal increase was calculated by expressing signals as percentage of the sample undergoing 15 min CXCL12 treatment (Figure 2H,I). After CXCL12 treatment, we observed a rapid loss of the signal produced by anti-S341-S352 and a complementary rapid increase of the signal produced by anti-pS346/347 (Figure 2A,B,G). Comparison of the signals obtained with the phospho-selective antibodies revealed that maximal phosphorylation was reached already after 5 min at S346/347 and at S324/325 (Figure 2I). The onset of phosphorylation appeared to be slightly faster at S346/347 than at S324/325 (Figure 2I). In contrast, full S338/339 phosphorylation required at least 15 min (Figure 2H). After 30 min CXCL12, phosphorylation at S338/339 was only slightly increased as compared with 15 min CXCL12 and phosphorylation at the other two sites was unchanged as compared with 15 min CXCL12 (not shown).

### Different Dephosphorylation Kinetics at S324/325, S338/339, and S346/347

**Figure 3:**
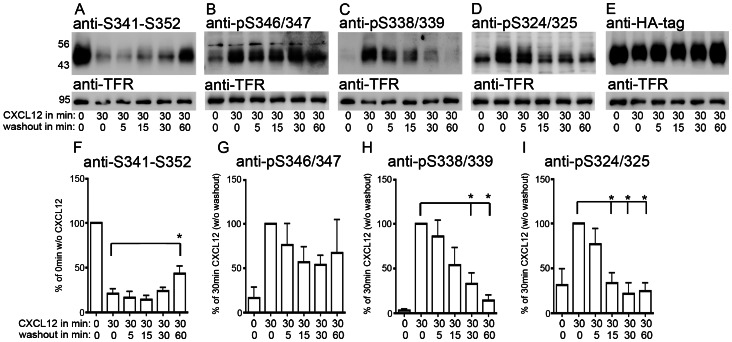
Long-lasting phosphorylation at S346/347. **A–E**, Stably CXCR4-transfected HEK293 cells were stimulated with CXCL12 for 30 min, washed with acidic buffer, and incubated with AMD3100-supplemented medium for the indicated intervals. Aliquots of the lysates were detected in five immunoblots using the indicated antibodies. The HA-tag and the endogenous transferrin receptor (TFR) were detected as loading controls. **A**,**B**, Slow recovery of the anti-S341-S352 signal corresponds to long-lasting phosphorylation at S346/347. **C**,**D**, Phosphorylation at S338/339 and S324/325 is rapidly reversed after CXCL12 washout. **E**, Detection with anti-HA demonstrates similar CXCR4 levels in the different samples. **F–I**, Using densitometry, the signal ratio versus anti-HA was determined for the four phospho-sensitive antibodies. Non-stimulated samples were set as 100% in *F* and samples undergoing 30 min CXCL12 stimulation were set as 100% in *G–I*. While there is little dephosphorylation at S346/347 (F,G**)**, major dephosphorylation occurs already 15 min after CXCL12 washout at S338/339 and S324/324 (H,I). Data represent mean+SEM from 4–5 independent experiments. *: p<0.05; 1way ANOVA followed by Dunnett´s post-test vs. the 30 min CXCL12/0 min washout group.

Dephosphorylation kinetics were studied after 30 min CXCL12 stimulation. The extended stimulation period was used to ensure maximum phosphorylation. The CXCL12 stimulus was ended by an acidic wash followed by 5, 15, 30, and 60 min ligand-free intervals in the presence of the CXCR4 antagonist AMD3100 (Figure 3A–D). Thus, the total experimental period was 90 min. Quantitative analyses were conducted like in the previous experiments by normalizing the signals of the phospho-sensitive antibodies with the corresponding signal of the anti-HA antibody (total CXCR4). We then expressed the signal of anti-S341-S352 as percentage of non-stimulated samples and the signals of the phospho-selective antibodies as percentage of the CXCL12-stimulated samples before washout (Figure 3F–I). This showed that the CXCL12-induced signal reduction of the anti-S341-S352 antibody recovered only partially during the 60 min washout period (Figure 3A,F), suggesting long-lasting phosphorylations in the C terminus. Indeed, the CXCL12-induced phosphorylation at S346/347 declined only slightly during the washout period (Figure 3B,G). In contrast, CXCL12-induced phosphorylation at both S338/339 and S324/325 was largely removed within 15 min after the washout (Figure 3C,D,H,I). Thus, CXCL12-induced phosphorylation at S346/347 is long-lasting whereas phosphorylation at S338/339 and S324/325 is transient.

It has been reported that extended CXCL12 stimulation (≥180 min) causes CXCR4 degradation in HEK293 cells [18]. To rule out that reduced signal intensity of the phospho-selective antibodies after the CXCL12 stimulation/washout period (90 min) was due to CXCR4 degradation, we compared the levels of total CXCR4 (anti-HA) and endogenous TFR in HEK293 cells undergoing 90 min CXCL12 stimulation. Consistent with the reported data [18], we found no significant CXCR4 degradation after 90 min CXCL12 treatment (not shown). In contrast, we readily observed CXCR4 degradation after long-term (overnight) CXCL12 treatment [35].

### GRK2 and GRK3 Mediate CXCL12-promoted Phosphorylation at S346/347

**Figure 4:**
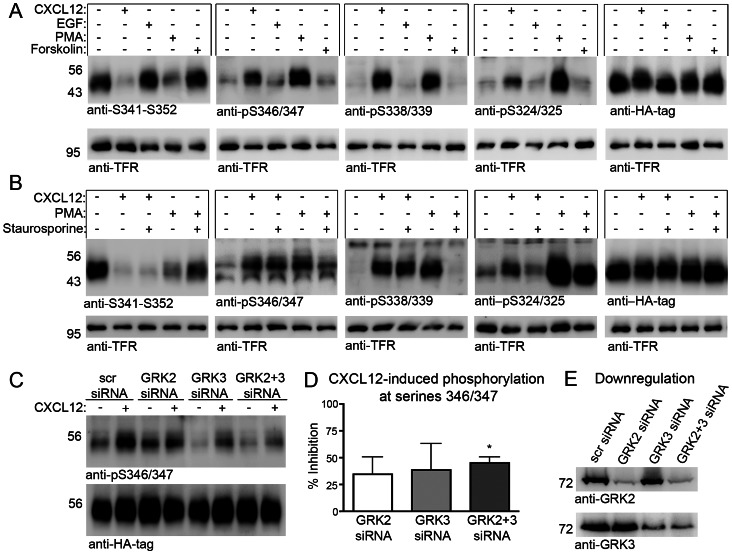
Heterologous and homologous phosphorylation of CXCR4. **A**, Stably CXCR4-transfected HEK293 cells were stimulated for 30 min as indicated to produce maximal effects. Note that CXCL12 and PMA but not EGF or forskolin induce CXCR4 phosphorylation. **B**, When indicated, cells were pre-incubated for 30 min with the non-selective PKC inhibitor staurosporine and stimulated for 15 min with CXCL12 or PMA in the presence or absence of staurosporine. Staurosporine reduces CXCL12-promoted phosphorylation at S324/325 and reduces PMA-induced phosphorylation at all three sites. **A**,**B**, Images are representative for three independent experiments yielding similar results. **C**,**D**, Cells were pre-treated for 72 h with control siRNA (scr) or siRNAs for GRK2 and GRK3 as indicated. Lysates were generated 15 min after CXCL12 stimulation and detected in an immunoblot with anti-pS346/347 and anti-HA. Combined knockdown of GRK2 and GRK3 reduces the CXCL12-promoted signal increase of anti-pS346/347 indicating attenuated phosphorylation at S346/347. **D**, Quantification of 3 independent experiments analyzing the effect of GRK2 and GRK3 knockdown on CXCL12-promoted S346/347 phosphorylation. *: p<0.05; Student's t test vs. scr-group. **E**, Western Blot analysis of the siRNA-mediated knockdown of GRK2 and GRK3.

Stimulus-dependent phosphorylation of GPCRs is mediated by multiple kinases including protein kinase A (PKA), protein kinase C (PKC), and G protein coupled receptor kinases (GRKs) [3]. Site-specific CXCR4 phosphorylation has been documented in response to CXCL12, PMA, and EGF and involves PKC and various GRKs [13], [14]. Because little information is available for S346/347 phosphorylation, we used anti-pS346/347 to compare effects of CXCL12, EGF, PMA, and the adenylyl cyclase activator forskolin at this site. For reference, phosphorylation was studied with anti-S341-S352, anti-pS338/339 and anti-pS324/325 in parallel. While both CXCL12 and PMA induced robust phosphorylation at the three sites, forskolin and EGF had virtually no effect (Figure 4A). Increased phosphorylation of the MAP kinases Erk1/2 confirmed successful EGF-stimulation (not shown).

We then used the PKC inhibitor staurosporine to test the contribution of PKC to the CXCL12- and PMA-induced CXCR4 phosphorylation (Figure 4B). This showed that the inhibitor had virtually no effect on the CXCL12-induced phosphorylation at S346/347 and S338/339 while it caused a strong reduction at S324/325. PMA-induced phosphorylation was reduced by staurosporine at all three sites (Figure 4B). Previous studies identified GRK6 in homologous phosphorylation at S324/325, S330, and S338/339 and proposed a role for GRK3 in homologous phosphorylation at the extreme C terminus [14], [38]. Using an established siRNA approach [36] we now demonstrate that CXCL12-induced phosphorylation at S346/347 was reduced after combined GRK2/GRK3 knockdown (45%) as compared with non-silencing control siRNA (Figure 4C,D). After single knockdown of GRK2 and GRK3, S346/347-phosphorylation tended to be reduced but the effect was not statistically significant. Thus, homologous phosphorylation at CXCR4 residues S346/347 is PKC-independent and mediated by GRK2/3.

### CXCL12-induced Phosphorylation at S324/325 and S338/339 is Reduced in WHIM Syndrome-associated CXCR4 Mutant Receptors

**Figure 5:**
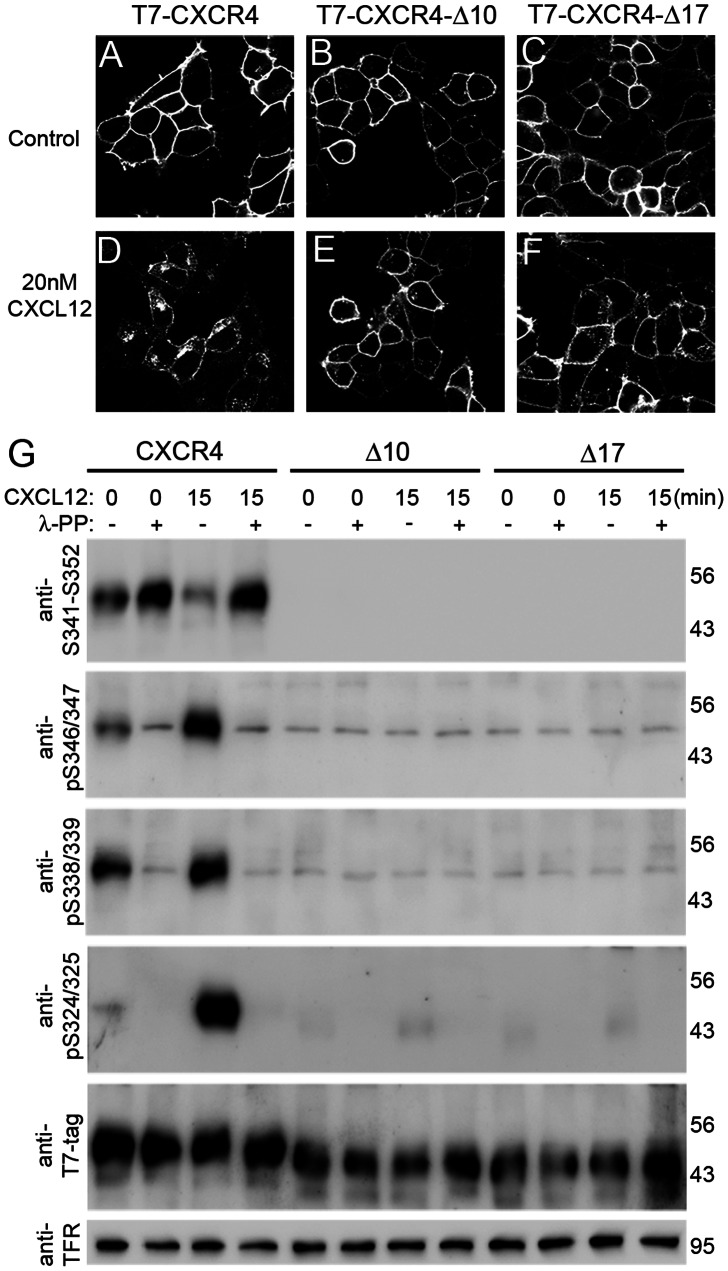
Hierarchical phosphorylation of CXCR4 C-terminal sites. HEK293 cells were transiently transfected with N-terminal T7 epitope-tagged wildtype CXCR4 (T7-CXCR4) and C-terminal deletion mutants lacking the last 10 and 17 residues (T7-CXCR4-Δ10, T7-CXCR4-Δ17). **A–F**, Surface receptors were pulse-labeled with anti-T7 antibody in live cells before cells received vehicle (A–C) or CXCL12 (D–F) for 30 min. Cells were fixed and permeabilized before immunofluorescent detection of anti-T7. In the absence of CXCL12 all three receptors are targeted to the plasma membrane showing little internalization (A–C). CXCL12 causes strong internalization of T7-CXCR4 (D) but only weak internalization of the C-terminal truncated receptors (E,F). **G**, Western blot analyses of CXCL12-induced phosphorylation of T7-CXCR4, T7-CXCR4-Δ10, and T7-CXCR4-Δ17 (CXCR4, Δ10, Δ17). HEK293 cells were transiently transfected with the indicated constructs and harvested before or 15 min after CXCL12 stimulation. Aliquots of the lysates were dephosphorylated using Lambda-Protein Phosphatase (λ-PP). Samples were loaded in four SDS gels, blotted, and immunodetected using anti-S341-S352, anti-pS346/347, anti-pS338/339, and anti-pS324/325. **T7-CXCR4:** In samples from non-stimulated transfectants λ-PP-treatment slightly enhances the signal of anti-S341-S352 and almost eliminates signals of the phospho-selective antibodies, indicating some constitutive CXCR4 phosphorylation. CXCL12 treatment causes a strong signal reduction of anti-S341-S352 and a strong signal increase of anti-pS346/347, anti-pS338/339, and anti-pS324/325. λ-PP-treatment of stimulated lysates fully restores the anti-S341-S352 signal and eliminates the signal of the phospho-selective antibodies. **T7-CXCR4-**Δ**10:** Deletion of the epitope eliminates specific signals of anti-S341-S352 and anti-pS346/347. There is virtually no constitutive phosphorylation at S338/339 and S324/325 in non-stimulated lysates. CXCL12-induced phosphorylation is not detected at S338/339 and strongly reduced at pS324/325 as compared with T7-CXCR4. **T7-CXCR4-**Δ**17:** Deletion of CXCR4 residues 336–352 eliminates specific signals of anti-S341-S352, anti-pS346/347, and anti-pS338/339. The mutant shows virtually no constitutive and no CXCL12-induced phosphorylation at S324/325. **Anti-T7-tag:** Stripping and detection with the anti-tag antibody reveals similar CXCR4 expression levels in all samples. **Anti-TFR:** Detection of endogenous transferrin receptor (TFR) in a stripped blot demonstrates equal loading. **G**, Results are representative for three independent experiments with similar results.

We then studied the influence of the extreme CXCR4 C terminus on phosphorylation at S324/325 and S338/339. To this end, we used two CXCR4 mutants with an N-terminal T7-tag and deletions of the last 10 and 17 C-terminal residues (CXCR4-Δ10, CXCR4-Δ17), which correspond to WHIM syndrome-associated mutations in the human CXCR4 gene [29], [39]. HEK293 cells were stably transfected with the mutants and a T7-tagged wildtype CXCR4. To validate expression of the three receptors, an immunocytochemical approach was used in which surface receptors were pulse-labeled at their extracellular N-terminal domain by applying anti-T7 antibody to live cells at 4°C (internalization-restrictive condition). Cells were washed and fixed after a 30 min 37°C interval (internalization-permissive condition) in the presence or absence of CXCL12. To permit the detection of antibody-labeled receptors both at the cell surface and inside the cells, we permeabilized the cells before applying Cy3-coupled secondary antibody. Mock-transfected cells included as controls proved negative (not shown). Using confocal microscopy, the three receptors were detected at the cell surface of non-stimulated cells (Figure 5A–C). Efficient CXCL12-promoted internalization was seen for wildtype CXCR4 (Figure 5D) but not for the truncated mutants (Figure 5E,F), which is consistent with a previous quantitative study employing ^125^I-labeled 12G5 anti-CXCR4 antibody [16].

CXCL12-induced phosphorylation was studied in transient transfectants 15 min after vehicle and CXCL12 stimulation. Since the phospho-selective antibodies produced some constitutive signals under these experimental conditions, we subjected aliquots of the lysates to Lambda-PP dephosphorylation to demonstrate phosphoselectivity of the signal (Figure 5G). CXCL12-induced phosphorylation was observed for S346/347, S338/339, and S324/325 of wildtype CXCR4. Surprisingly, deletion of the last 10 residues eliminated not only binding of the anti-S341-S352 and anti-pS346/347 antibodies, it also inhibited CXCL12-induced phosphorylation at S338/339 and S324/325. Similarly, deletion of the last 17 residues abolished not only antibody binding at the deleted region (S346/347 and S338/339), it also prevented CXCL12-induced phosphorylation at S324/S325.

### E343 and S346-S348 are Required for Efficient CXCL12-promoted Phosphorylation at S324/325 and S338/339, CXCR4 Internalization, and CXCR4 Desensitization

**Figure 6:**
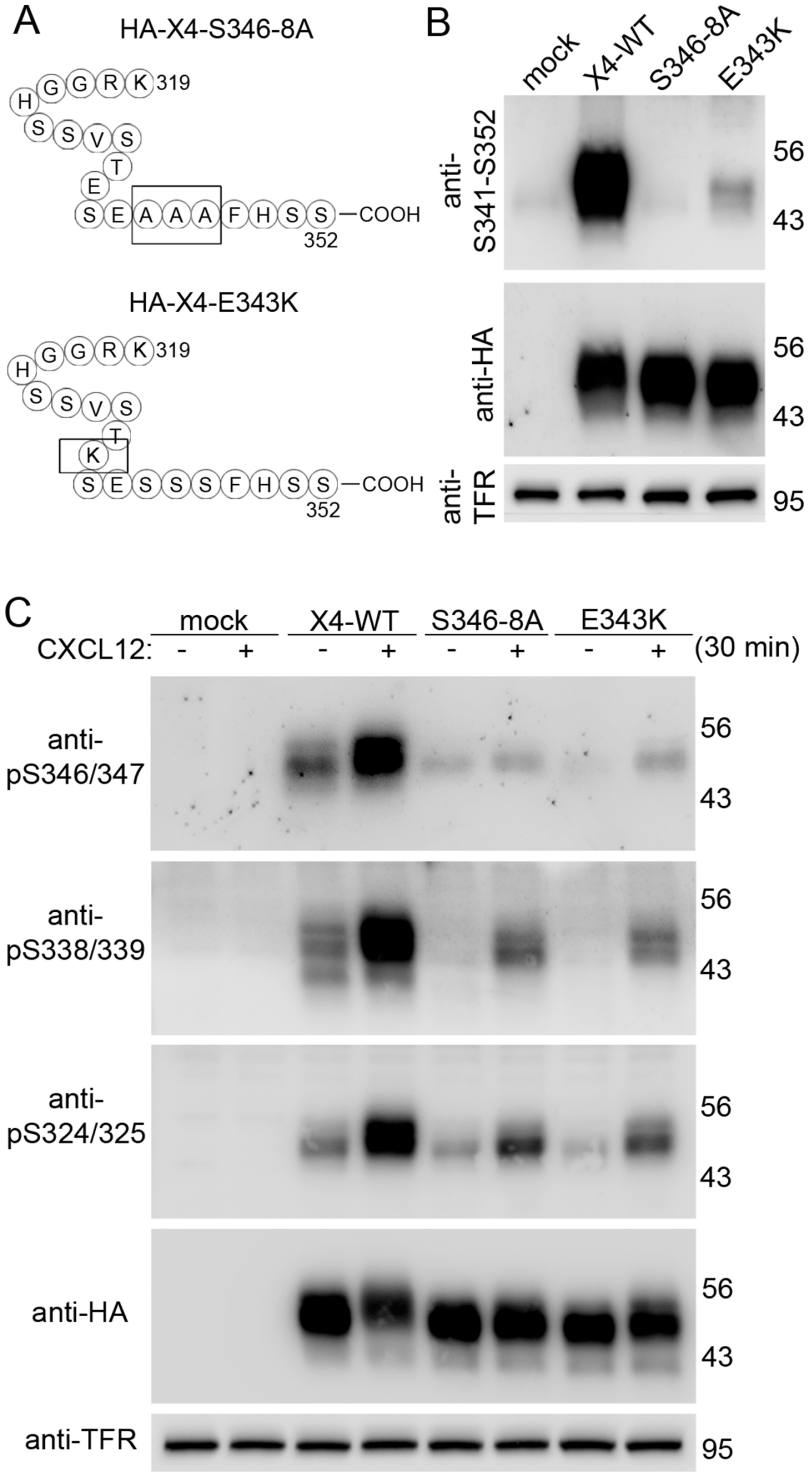
Decreased phosphorylation at S338/339 and S324/325 in CXCR4-S346-8A and CXCR4-E343K mutants. **A**, Schematic representation of the HA-CXCR4-S346-8A and HA-CXCR4-E343K mutants. **B**,**C**, HEK293 cells were transfected with empty vector (mock) and the indicated CXCR4 mutants. **B**, The anti-S341-S352 antibody recognizes CXCR4-E343K only slightly and does not bind to CXCR4-S346-8A. **C**, Cells were stimulated with 20 nM CXCL12 for 30 min. Aliquots of the lysates were detected in four immunoblots using the indicated antibodies. Blots were stripped and reprobed with anti-HA and anti-transferrin receptor (TFR) to control for equal CXCR4 protein and loading. **C**, CXCR4 mutants are not phosphorylated at S346/347 after CXCL12-stimulation. Constitutive and induced phosphorylation at S338/339 and S324/325 is strongly diminished as compared with wildtype CXCR4. **B**,**C**, Results are representative for three independent experiments with similar results.

Having shown that the CXCL12-promoted phosphorylation at S346/347 precedes that at S324/325 and S338/339 and that phosphorylation at S324/325 and S338/339 is severely reduced when the last ten CXCR4 residues are absent, we hypothesized that phosphorylation at residues S346-S348 is obligate for efficient phosphorylation to occur at the other sites. To test this hypothesis, we generated a HA-tagged S346-348A mutant (HA-CXCR4-S346-8A, Figure 6A). Given that GRK3 contributes to the CXCL12-promoted phosphorylation at S346/347 and that GRK3 prefers acidic residues N-terminal to the phospho-site [40], we also generated a HA-tagged E343K mutant (HA-CXCR4-E343K, Figure 6A), which corresponds to a recently discovered WHIM syndrome-associated mutation [30]. The introduced amino acid changes eliminated (S346-8A) and impaired (E343K) binding of the anti-S341-S352 antibody (Figure 6B). The anti-pS346/347 antibody did not recognize non-stimulated and CXCL12-stimulated HA-CXCR4-S346-8A, which confirms specificity of the antibody (Figure 6C).

To study the effect of the introduced mutations on CXCR4 phosphorylation, we then examined lysates from CXCL12-stimulated cells using the phospho-selective antibodies (Figure 6C). Cells were examined after 30 min CXCL12 exposure to ensure maximal phosphorylation. Interestingly, elimination of the S346-S348 motif caused a strong reduction of the CXCL12-promoted phosphorylation at S338/339 and S324/325 (Figure 6C). In the E343K mutant, CXCL12-promoted phosphorylation was severely reduced at S346/347, S338/339, and S324/325 (Figure 6C).

**Figure 7:**
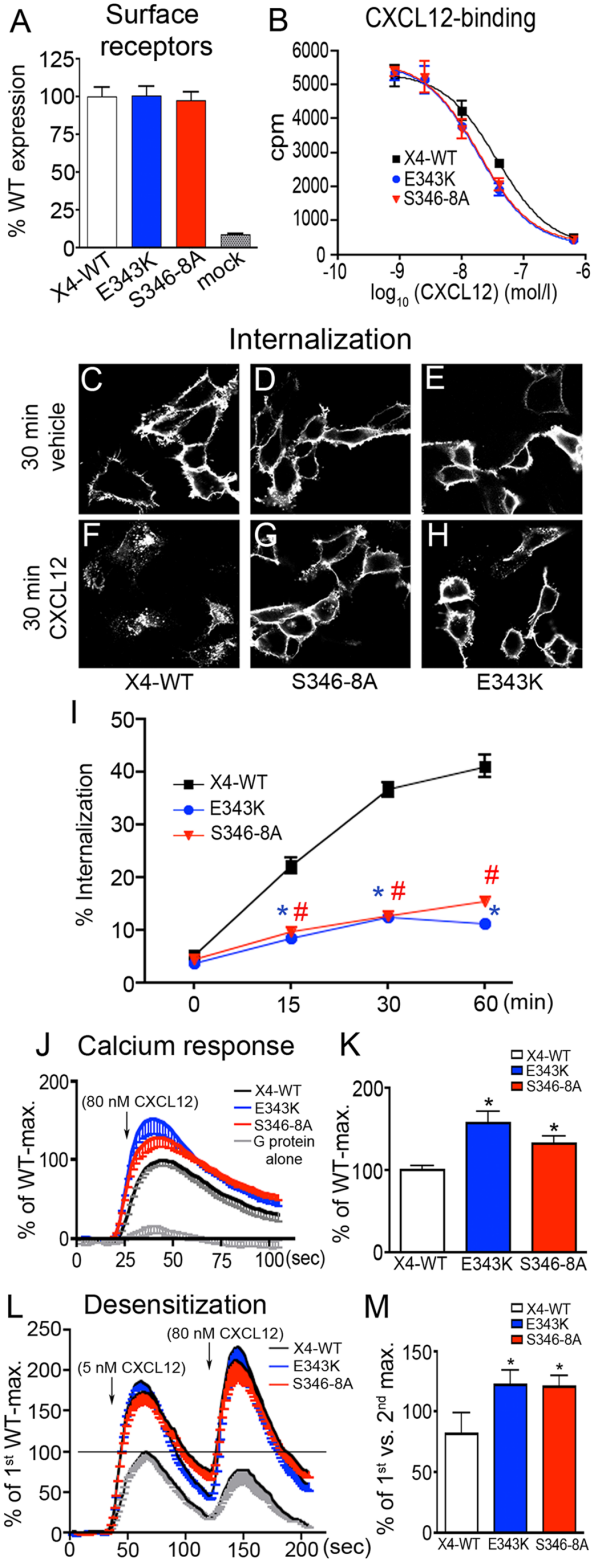
Functional characterization of HA-CXCR4-S346-8A and HA-CXCR4-E343K mutants. **A–M**, Wildtype CXCR4 (X4-WT) and the mutants were transiently transfected in HEK293 cells. **A**, Surface receptor levels of the transfectants were analyzed by ELISA using anti-HA antibody. Results are given as percent of wildtype (WT) levels and represent mean+SEM from two independent experiments with 3 to 5 repeats. **B**, Homologous radioligand competition binding using ^125^I-CXCL12 (25 pM) and increasing concentrations of unlabeled CXCL12. The three CXCR4 variants have comparable CXCL12 binding properties (WT, 37 nM; E343K and S346-8A, 18 nM). **C–H**, Surface receptors were pulse-labeled with anti-HA antibody applied to live cells at 4°C. Confocal images show the subcellular localization of labeled receptors after a 30 min interval at 37°C in absence of ligand (C–E) or in the presence of CXCL12 (F–H). Cells transfected with wildtype CXCR4 show major internalization after CXCL12 stimulation (F). In cells transfected with mutant receptors, the majority of the staining remains plasma-membrane associated even after 30 min CXCL12 stimulation (G,H). **I**, Internalization kinetics of surface receptors labeled with ^125^I-CXCL12. Transfected cells were loaded with ^125^I-CXCL12 at 4°C (pulse), washed, and lysed immediately (starting value) or incubated for the indicated time intervals at 37°C and lysed after an acidic wash (chase). Amounts of internalized radioligand are given as percent of the starting value. Results represent mean ± SEM from two independent experiments with 4 repeats each. Internalization of radioligand-labeled mutant receptors is significantly reduced. *, #: p<0.05; 2way ANOVA vs. wildtype CXCR4. **J–M**, G protein coupling of wildtype CXCR4 and of the mutants. CXCR4 constructs were co-transfected with the 16z44 chimeric G protein and changes in Ca^2+^_i_ were monitored. **J**,**K**, Single stimulation with 80 nM CXCL12 reveals an increased peak height in cells expressing the mutant CXCR4 receptors. Data were normalized to mean of the wildtype maximum and plotted as averaged traces with SEM. **K**, Quantitative analysis of the average peak height. *: p<0.05; Student´s t tests vs. wildtype CXCR4. **L**,**M**, Averaged traces with SEM showing two subsequent stimulations with CXCL12, using 5 nM and 80 nM concentrations. **L**, Data was normalized to the mean of the first maximum of wildtype CXCR4. **M**, For analysis of homologous desensitization, the second peak was expressed as percent of the first peak for each trace before mean values+SEM of the peak ratios were calculated. Increased ratios in the mutants indicate reduced desensitization. *: p<0.05; Student´s t test vs. wildtype CXCR4.

We then asked if the S346-8A mutation perturbs CXCR4 function like CXCR4 mutations causing WHIM syndrome and compared the S346-8A mutant with the E343K WHIM mutant and wildtype CXCR4. First, we determined the receptor levels at the cell surface using an ELISA and a transient transfection protocol (the same protocol was used also in the subsequent experiments). After pulse labeling of the extracellular HA-tag with anti-HA antibody in live cells at 4°C and several washes, cells were fixed and detected with a peroxidase-conjugated secondary antibody and a peroxidase-driven color reaction. The assay showed similar labeling intensities for wildtype CXCR4, E343K-CXCR4, and S346-8A-CXCR4 (Figure 7A). Mock-transfected cells included as controls produced only background signals (Figure 7A). IC_50_ values determined in homologous competitive radioligand-binding experiments showed that the CXCR4 affinity for CXCL12 was not affected by the S346-8A or the E343K mutation (Figure 7B). CXCL12-promoted CXCR4 internalization was then analyzed qualitatively by immunocytochemistry (Figure 7C–H) and quantitatively by radioligand-labeling (Figure 7I). After pulse-labeling of surface receptors by anti-HA at 4°C and a 30 min CXC12-free interval, receptors were seen almost exclusively at the cell surface of the different transfectants (Figure 7C–E). Application of CXCL12 during the 30 min interval caused robust internalization of wildtype CXCR4 (Figure 7F) but little internalization of S346-8A-CXCR4 and E343K-CXCR4 (Figure 7G,H). Consistently, wildtype CXCR4 receptors that were pulse-labeled by radioligand at 4°C (internalization-restrictive condition) were rapidly internalized after being transferred to 37°C (internalization-permissive condition) while CXCR4-S346-8A and CXCR4-E343K showed little internalization (Figure 7I).

G protein coupling of CXCR4 was examined in calcium mobilization experiments using FLIPR technology. The CXCR4 variants were co-transfected with a 16z44 G protein chimera that was engineered to link G_i_-coupled receptors to the calcium pathway [37]. CXCL12 (80 nM) evoked only slight calcium responses in the absence of recombinant CXCR4 but produced strong responses when CXCR4 was co-transfected with 16z44 (Figure 7J). Analysis of the peak maxima showed that CXCR4-E343K and CXCR4-S346-8A produced stronger calcium signals than wildtype CXCR4 (Figure 7K), suggesting that S346-8A represents a gain-of-function mutation like E343K [30].

Sequential stimulation was applied to determine homologous CXCR4 desensitization. When CXCL12 (80 nM) was administered 180 s after a precedent 80 nM stimulus, it evoked only a small calcium response irrespective of the CXCR4 variant examined (not shown). To circumvent major CXCR4 desensitization, we reduced the CXCL12 concentration in the pre-stimulus to 5 nM. Normalization of the traces with the mean of the first maximum of wildtype CXCR4 showed that the calcium responses to both the first 5 nM stimulus and to the second 80 nM stimulus were larger with CXCR4-E343K and CXCR4-S346-8A than with wildtype CXCR4 (Figure 7L), which confirms that the E343K and the S346-8A mutations cause a gain-of-CXCR4-function. Desensitization was calculated for the three CXCR4 variants by dividing the second maximum of each trace by its first maximum. The average ratio of first and second maximum was significantly larger with CXCR4-E343K and CXCR4-S346-8A than with wildtype CXCR4 (Figure 7M), suggesting that the pre-stimulus caused less desensitization of the mutant receptors than of wildtype CXCR4.

## Discussion

Using novel and established phospho-selective antibodies for the CXCR4 C-terminal sites S324/325, S338/339, and S346/347, we provide evidence that S346/347 represents a major C-terminal phosphorylation site. Upon CXCL12 stimulation, S346/347 reached maximal phosphorylation faster than S338/339 and S324/325. Deletion of S346-S348 reduced CXCL12-promoted phosphorylation at S324/325 and S338/339, caused a gain-of-CXCR4-function and reduced receptor internalization and desensitization. Our findings indicate hierarchical organization of the multiple phosphorylation events taking place at the CXCR4 C-terminal domain and help explain why mutations in the CXCR4 gene which cause small alterations at the extreme C terminus can severely affect CXCR4 regulation and signaling.

### The anti-S341-S352 Antibody Recognizes Only the Non-phosphorylated CXCR4 C Terminus

We established that the rabbit monoclonal anti-S341-S352 antibody UMB-2 is highly selective for CXCR4 and that the antibody recognizes only the non-phosphorylated C terminus. Immunoblots with lysates from CXCR4-overexpressing HEK293 cells showed that 15 minutes CXCL12 stimulation were sufficient to trigger an almost complete loss of UMB-2 signal. The finding that dephosphorylation fully restored the UMB-2 signal indicates that one or several of the potential phosphosites in the UMB-2 epitope (S341, T342, S344, S346-S348, and S351-S352) become quantitatively phosphorylated upon CXCL12 exposure. Thus, by detecting dephosphorylated and non-dephosphorylated aliquots of a sample with UMB-2 one can visualize total and non-phosphorylated CXCR4. The difference between the two signals reflects C-terminal CXCR4 phosphorylation and can serve as an indicator for CXCR4 activation. UMB-2 readily detects low endogenous CXCR4 levels in HEK293 cells and brain tissue [34] whereas our phospho-selective antibodies failed to produce any signals under these conditions. Because the UMB-2 epitope is conserved in human, rat and mouse, UMB-2 is a sensitive tool to study expression and activation of endogenous CXCR4 in cell lines and tissue samples.

### Differential Regulation of Phosphosites S324/325, S338/339, and S346/347

Our dot blot analyses showed that phosphorylation at the S346/347 site is sufficient to prevent UMB-2 binding. Given that UMB-2 fails to recognize lysates from CXCL12-stimulated cells, we reasoned that the S346-S348 motif might contain a major CXCR4 phosphorylation site. To test this assumption, we developed a phospho-selective anti-pS346/347 antibody. Using this tool, we showed that this site is indeed rapidly phosphorylated upon CXCL12 stimulation. CXCL12-promoted phosphorylation at S346/347 was mediated by GRK2/3 and occurred in a PKC-independent manner. This property renders S346/347 distinct from previously identified phosphosites in the CXCR4 C terminus, which include S324/325, S330, and S338/339 [13], [14]. Moreover, phosphorylation at S346/347 peaked slightly faster than PKC- and GRK6-mediated phosphorylation at S324/325 and considerably faster than GRK6-mediated phosphorylation at S338/339. Previously, comparison of the GRK6 sites S330 and 339 showed that phosphorylation kinetics are even slower at S330 than at S339 [14]. Collectively, these findings suggest that CXCL12 promotes phosphorylations first via GRK2/3 at S346/347 and via GRK6 and PKC at S324/325 before the GRK6 sites S338/339 and S330 are used.

Another distinguishing feature of S346/347 phosphorylation is its permanent nature: whereas S324/325 and S338/339 were rapidly dephosphorylated after CXCL12 washout, major dephosphorylation was not observed for S346/347 during the investigated 60 min ligand-free interval. Ligand-induced phosphorylation of GPCRs typically induces ß-arrestin/receptor interaction [41]. Thus, long-term phosphorylation at S346/347 might favor a stable CXCR4/ß-arrestin complex, a characteristic of receptors which have been categorized as ´class B ´ receptors [42]. For instance, in the case of the vasopressin V2 receptor (V2R), which is a prototypical class B receptor, a cluster of three serine residues close to the C terminus mediates formation of a stable V2R/ß-arrestin complex and prevents rapid dephosphorylation, resensitization, and recycling of the receptor [42], [43]. Evidence has been provided that conversion of CXCR4 serine residues 346-348 and 351/352 into alanines eliminates CXCR4/ß-arrestin interaction whereas alanine conversions at the GRK6 sites facilitate CXCR4/ß-arrestin interaction [14]. Thus, CXCR4 might show class A and class B properties depending on which C-terminal phosphorylation sites are used. However, CXCR4/ß-arrestin interactions involve not only the C terminus but occur also via a SHSK motif in the third intracellular loop [44].

### Hierarchical CXCR4 Phosphorylation and WHIM Syndrome

Most WHIM syndrome patients carry heterozygous mutations in the CXCR4 gene which eliminate the last 10 to 19 residues of the C terminus [45], [46], [47]. Current concepts for the genesis of WHIM syndrome focus on disturbances in the CXCR4 C-terminal phosphorylation and ß-arrestin recruitment events [29], [39]. A particular feature of the WHIM syndrome CXCR4 mutant receptors is a delay in the receptor sequestration that follows CXCL12 exposure, and substantial evidence has been presented that WHIM receptors are gain-of-function mutants causing prolonged signaling [30], [44], [47], [48], [49]. It therefore comes as a surprise that CXCR4 serine residues 324 and 325, which are critical for CXCL12-promoted CXCR4 internalization and degradation [16], [17], [39], are intact in all described WHIM mutant receptors. Given our finding that residues 343–352 are required for efficient CXCL12-promoted phosphorylation at S324/325 and S338/339 provides an explanation for this apparent discrepancy.

But how do WHIM patients lacking CXCR4 mutations [29] and the recently identified E343K WHIM mutation in the CXCR4 receptor [30] fit into the concept? The fact that E343K is a charge-changing substitution N-terminal to S346/347 provides a clue to this question. It is generally accepted that GRKs 2 and 3 prefer acidic residues N-terminal to the phosphorylation site [40]. Given our observation that S346/347 is a GRK2/3 site, it seems reasonable to assume that E343K impairs GRK2/3-mediated phosphorylation at S346/347. Since efficient phosphorylation at S324/325 and S338/339 requires phosphorylation at S346/347, it is conceivable that impaired GRK2/3-mediated S346/347 phosphorylation in the E343K mutant affects also phosphorylation events at the more N-terminal GRK6 sites. Reports that GRK3 plays a critical role in regulating CXCR4 signaling and that GRK3 activity is reduced in WHIM patients with normal CXCR4 receptors [38] lend support to this view.

### Conclusions

Our studies in HEK293 cells show that serine residues 346 and 347 constitute a major CXCR4 phosphorylation site. Elimination of the S346-S348 cluster severely impairs phosphorylation at the intact C-terminal sites S324/325 and S338/339. We suggest that phosphorylation events at the CXCR4 C terminus occur hierarchically requiring initial phosphorylation at S346/347. These results identify a novel structural determinant of CXCR4 regulation and help decipher how mutations affecting the CXCR4 C terminus lead to signaling perturbances and disease.

## Supporting Information

Figure S1 **Dot blot analysis of phospho-sensitive CXCR4 antibodies.** **A**, Schematic representation of CXCR4 C-terminal residues 322–352. Sequences of peptides #1 - #3 used in the dot blot are underlined. Serine residues 324/325, 338/339, and 346/347 used for phosphorylation in the phosphopeptides are highlighted. **B**, Decreasing amounts (2–0.125 µg) of non-phospho and phosphopeptides #1 - #3 were blotted onto 4 PVDF membranes and detected with anti-S341-S352, anti-pS346/347, anti-pS338/339, and anti-pS324/325 as indicated. Anti-S341-S352 detects the S341-S352 epitope only when serine residues 346 and 347 are not phosphorylated. The phosphoantibodies recognize their phosphorylated epitopes with high selectivity showing little crossreactivity.
(TIF)
