# Supplementary figures and images for: Hierarchical Organization of Multi-Site Phosphorylation at the CXCR4 C Terminus
Source: PLoS One. 2013 May 29;8(5):e64975. doi: 10.1371/journal.pone.0064975 (PMC3666969; doi:10.1371/journal.pone.0064975)

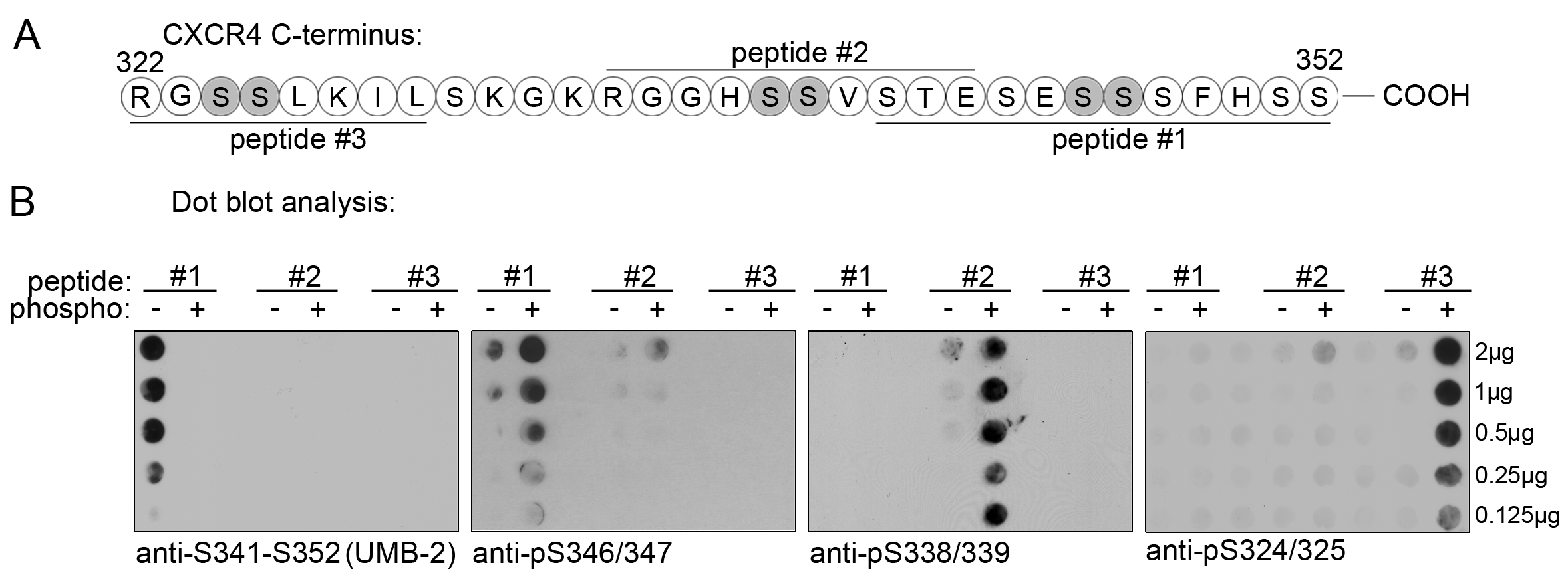

Supplement: Figure S1 — Dot blot analysis of phospho-sensitive CXCR4 antibodies. A, Schematic representation of CXCR4 C-terminal residues 322–352. Sequences of peptides #1 - #3 used in the dot blot are underlined. Serine residues 324/325, 338/339, and 346/347 used for phosphorylation in the phosphopeptides are highlighted. B, Decreasing amounts (2–0.125 µg) of non-phospho and phosphopeptides #1 - #3 were blotted onto 4 PVDF membranes and detected with anti-S341-S352, anti-pS346/347, anti-pS338/339, and anti-pS324/325 as indicated. Anti-S341-S352 detects the S341-S352 epitope only when serine residues 346 and 347 are not phosphorylated. The phosphoantibodies recognize their phosphorylated epitopes with high selectivity showing little crossreactivity. (TIF) [file pone.0064975.s001.tif]
